# Prevalence of Neurodivergence in Neuropsychiatric Conditions

**DOI:** 10.1192/bjo.2025.10238

**Published:** 2025-06-20

**Authors:** Grace Fearnehough, Bruce Tamilson, Andrea De Angelis

**Affiliations:** 1 South West London and St George’s Mental Health NHS Trust, London, United Kingdom; 2 St George’s University of London, London, United Kingdom; 3 St George’s University Hospital, London, United Kingdom; 4 Kingston and Richmond NHS Trust, London, United Kingdom

## Abstract

**Aims:** The increasing recognition of neurodivergent conditions within healthcare frameworks highlights the necessity for better understanding and management in neuropsychiatric settings. These conditions often overlap with complex neuropsychiatric diagnoses, complicating both diagnosis and treatment. This study aims to investigate the prevalence and co-occurrence of neurodivergent conditions and traits among patients with neuropsychiatric conditions.

**Methods:** A descriptive, quantitative cross-sectional study was conducted at a tertiary regional neuropsychiatric outpatient clinic in London. Participants included 166 consecutive patients, assessed using the Comprehensive Autistic Trait Inventory (CATI) and the Adult ADHD Self-Report Scale (ASRS-v1.1), with demographic characteristics considered.

**Results:** The study identified significantly higher rates of ASD and ADHD traits among patients with various neuropsychiatric conditions, particularly in those diagnosed with Functional Neurological Disorder (FND). Statistical analyses reinforced the heightened prevalence of these traits compared with general population estimates.

**Conclusion:** The findings indicate a higher-than-expected prevalence of neurodivergent conditions in patients with Functional Neurological Disorder. Enhanced early identification and tailored treatment approaches are crucial for improving clinical outcomes and patient experiences in neuropsychiatric settings.

